# Hiperoxalúria Primária com Acometimento Cardíaco: Uma Série de Casos

**DOI:** 10.36660/abc.20250574

**Published:** 2026-04-01

**Authors:** Diane Xavier de Ávila, Christine Zomer Dal Molin, Josué dos Santos Barbosa, Laís Corrêa de Carvalho, João Padula Rocha, Maria Júlia Hallack Moura, Davi Orli Machado Grüdtner, Raquel Bittencourt

**Affiliations:** 1 Complexo Hospitalar de Niterói Niterói RJ Brasil Complexo Hospitalar de Niterói, Niterói, RJ – Brasil; 2 Universidade Federal de Santa Catarina Araranguá SC Brasil Universidade Federal de Santa Catarina, Araranguá, SC – Brasil; 3 Serviço de Verificação de Óbitos de Criciúma Criciúma SC Brasil Serviço de Verificação de Óbitos de Criciúma, Criciúma, SC – Brasil

**Keywords:** Doenças Cardiovasculares, Falência Renal Crônica, Hiperoxalúria Primária

## Introdução

**Figura 1:**
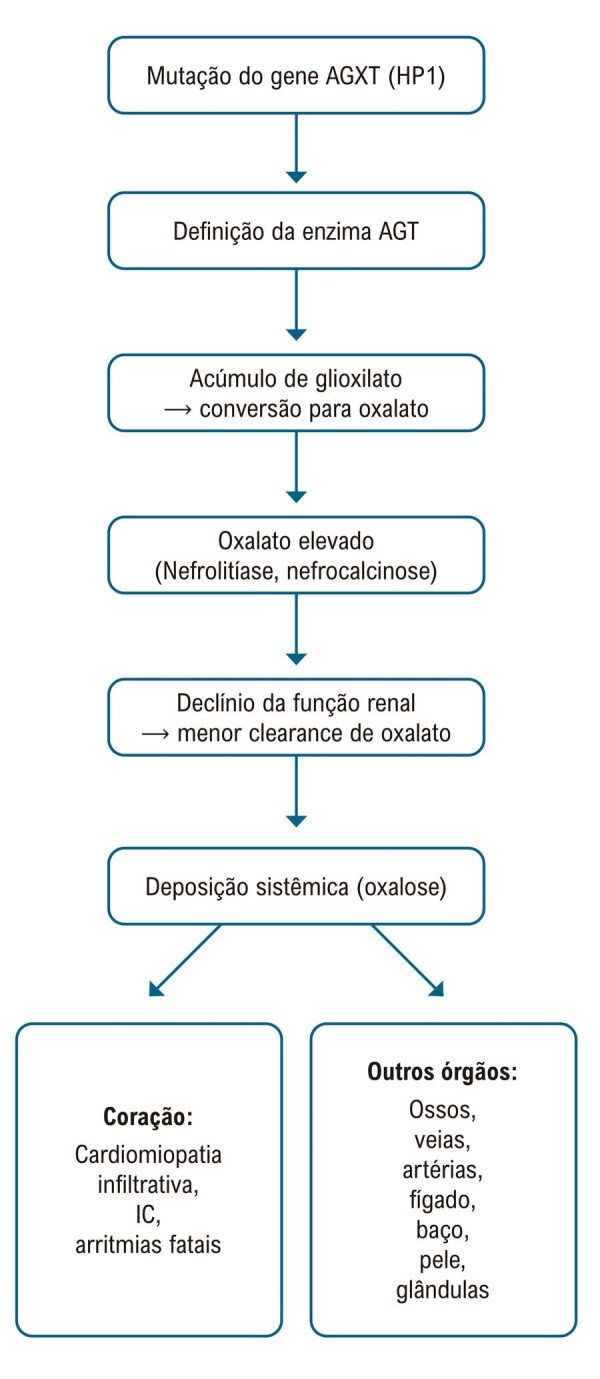
– Fisiopatologia e manifestações da HP1.

A hiperoxalúria primária tipo 1 (HP1) é uma doença autossômica recessiva rara causada por uma mutação no gene AGXT, que codifica a enzima responsável pela metabolização do glioxilato. Essa disfunção leva ao acúmulo de glioxilato e a sua conversão para oxalato, resultando em níveis séricos aumentados de oxalato, deposição tecidual e lesão de órgãos, especialmente rins e coração.^1 - 3^ O envolvimento cardíaco pode progredir para insuficiência cardíaca (IC) e arritmias fatais.^3 , 4^ Uma visão geral da fisiopatologia da HP1 está ilustrada na Figura 1 . Este artigo apresenta dois pacientes com HP1, oxalose e as consequências cardíacas desta doença. Esta série de casos foi elaborada em conformidade com as diretrizes CARE,^5^ tendo sido submetida e aprovada pelo Comitê de Ética da Universidade Federal de Santa Catarina.

## Caso 1

Uma mulher de 19 anos, caucasiana, previamente diagnosticada com nefrocalcinose medular bilateral aos 3 anos, iniciou quadro de síndrome urêmica. Iniciou-se terapia de substituição renal (TSR) imediatamente. A hiperoxalúria e a nefrocalcinose prévia levantaram suspeita para HP1. A análise genética confirmou variantes bialélicas no AGXT: c.33del e p.Lys12Argfs*34.

O tratamento da paciente se limitou a uma abordagem conservadora, que incluiu manejo da IC, hemodiálise e piridoxina. O Lumasiran não estava disponível no sistema público de saúde. Os achados eletrocardiográficos revelaram ritmo sinusal com sobrecarga atrial esquerda (AE) e bloqueio divisional ântero-superior esquerdo. Ecocardiogramas mostraram câmaras cardíacas dilatadas, com importante dilatação biatrial e hipertrofia excêntrica do ventrículo esquerdo (VE) resultando em disfunção sistólica com fração de ejeção (FE) de 34,9% (Simpson), com espessura de parede posterior de 13 mm. Estudos de imagem também apontaram para regurgitamento leve de valvas aórtica, mitral e tricúspide. A concentração de peptídeo natriurético tipo B (BNP) estava acima de 500 pg/ml. O escore de Agatston era zero. Estudos de ressonância magnética cardíaca demonstraram hipocinesia miocárdica difusa, disfunção sistólica esquerda importante com FEVE de 28%, disfunção sistólica direita moderada (FE do ventrículo direito (VD) de 25%), valores elevados de T1 map e ausência de trombo atrial. Esses achados, especialmente os ecocardiográficos, sugerem cardiomiopatia infiltrativa, provavelmente relacionada a essa doença sistêmica.

**Figura 2:**
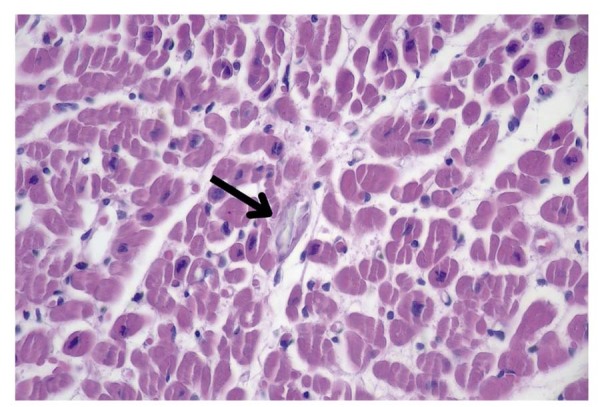
– Tecido miocárdico com oxalose. Coloração por H&E (40x).

Um ano depois de começar a TSR, a paciente evoluiu com morte súbita enquanto aguardava uma biópsia medular no hospital. Apesar do início imediato das manobras de ressuscitação cardiopulmonar, não foi possível reverter o quadro, culminando no óbito. A autópsia revelou embolia pulmonar tromboembólica, possivelmente relacionada à disfunção cardíaca pré-existente. A histopatologia mostrou atrofia miocárdica e depósitos de oxalato nos miócitos, territórios intersticiais e intravasculares. Os depósitos se estendiam ao pericárdio e ao endocárdio, sem inflamação ( Figuras 2 e 3 ).

**Figura 3:**
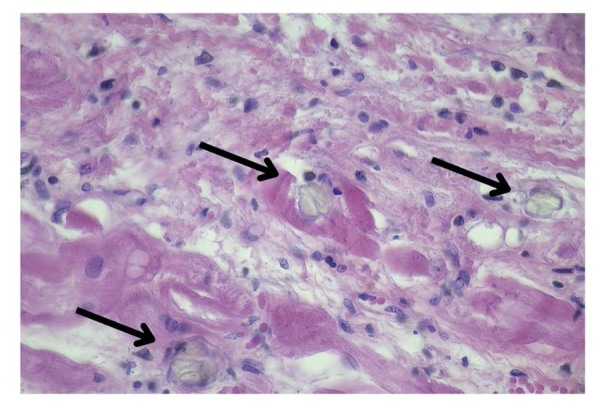
– Tecido miocárdico com oxalose. Coloração por H&E (40x).

## Caso 2

Um homem de 42 anos, irmão da paciente previamente descrita, não tinha histórico prévio de doença renal até os 35 anos, quando foi necessário iniciar TSR devido a níveis extremos de escórias nitrogenadas, anemia e hiperparatireoidismo. Exames de imagem revelaram rim direito com dimensões reduzidas, aumento de rim esquerdo e hidronefrose esquerda em decorrência de ureterolitíase, nefrolitíase bilateral e perda da diferenciação corticomedular. Uma cintilografia renal com DMSA evidenciou grave comprometimento crônico da função tubular renal. Devido ao histórico familiar, o diagnóstico de HP1 foi suspeitado e testes genéticos revelaram ambas as mutações patogênicas no gene AGXT: c.33del (p.Lys12Argfs*34) e c.508G>A (p.Gly170Arg). Investigações cardíacas futuras indicaram disfunção diastólica do VE, com função sistólica preservada do VE (74,9 %, Simpson), além de aumento do átrio direito (AD), sem evidências de disfunção valvar. A concentração do BNP foi de 875 pg/ml, e o nível sérico de oxalato, antes do início do tratamento, de 59 µmol/ml. Após cerca de dois anos, o paciente obteve autorização judicial para iniciar tratamento com Lumasiran, coberto pelo SUS (Sistema Único de Saúde).

## Discussão

HP1 é o tipo mais comum e severo das hiperoxalúrias primárias (HPs). É uma doença autossômica recessiva particularmente prevalente no norte de África e no Oriente Médio, dada a alta taxa de consanguinidade, e resulta da deficiência da enzima AGT, codificada pelo gene AGXT. Mais de 200 mutações do gene AGXT existem, mas a p.Gly170Arg e a c.33dupC são as mais frequentes.^1^

Apesar de o diagnóstico de HP1 ser fortemente considerado em pacientes com urolitíase recorrentes por oxalato de cálcio, nefrocalcinose e doença renal crônica (DRC) progressiva de etiologia desconhecida ou um histórico familiar de urolitíase e DRC, fatores como a apresentação heterogênea, sintomas inespecíficos e a dependência de testes genéticos para confirmação levam a um diagnóstico desafiador. Até 50% dos casos de HP1 são diagnosticados em estágios avançados de DRC, aumentando a mortalidade.^1^ Entretanto, o rastreio de familiares pode ajudar no diagnóstico precoce, tornando possível um tratamento bem sucedido.^1^

A variabilidade da idade de início da doença e da severidade da patologia é um desafio adicional em identificar a HP1. A manifestação inicial da doença pode ocorrer em todas as idades, mas tipicamente manifesta-se na infância, com nefrolitíase, nefrocalcinose e falência renal.^1^ Assim que o diagnóstico é feito, preconiza-se a avaliação da possibilidade de oxalose.

O acúmulo de oxalato de cálcio na urina causa nefrolitíase e nefrocalcinose. Com o declínio da função renal, o clearance de oxalato diminui, o que causa deposição sistêmica, especialmente no coração.^3 , 4^ Outros órgãos afetados são ossos, veias e artérias, fígado, baço, pele e glândulas.^6^

Essa fisiopatologia levou às alterações cardíacas do caso 1, que estavam em concordância com uma série de casos prévia.^4^ Neste estudo anterior, algumas alterações foram observadas: hipertrofia de VE, bloqueio de ramo esquerdo, bloqueio atrioventricular, disfunção valvar, FE reduzida, índice de massa de VE aumentado, dilatação AE, FEVD reduzida, hipertensão pulmonar, disfunção diastólica e processos infiltrativos. Todas as alterações descritas predispõem à IC e arritmias fatais. No mesmo estudo mencionado, pessoas com anormalidades cardíacas tinham creatinina sérica média de 7.16 (0,9-33.2) mg/dL, enquanto pessoas sem anormalidades tinham uma média de 1.93 (0,3-21.6). Além disso, 77,8% das pessoas com alterações cardíacas possuem DRC em estágio final.^4^

Estes dados destacam a importância de monitorar a função cardíaca na HP1, particularmente se função renal reduzida. A redução precoce do oxalato sérico pode prevenir envolvimento cardíaco, reiterando a necessidade do acesso aos tratamentos modernos, que serão discutidos abaixo.

Duas medicações para manejo de HP1 recentemente ganharam proeminência: Lumasiran e Nedosiran.^7^ O paciente do caso 2 está sob tratamento com Lumasiran pelos últimos 9 meses.

Apesar do alto custo destas medicações, elas reduzem significativamente a produção de oxalato. Lumasiran e Nedosiran inibem a produção hepática de oxalato por meio da terapia de interferência por RNA. O principal efeito adverso destes fármacos são reações de sítio de injeção (Lumasiran e Nedosiran) e dor/desconforto abdominal (Lumasiran),^8^ mas o paciente do caso 2 não sofreu nenhum efeito adverso.

Outra abordagem para a HP1 é a piridoxina, cuja responsividade deve ser testada em todos os pacientes com esta patologia (recomendação forte).^9^ Outras terapias, incluindo estiripentol, carbonato de lantânio e Oxalobacter formigenes carecem de evidências robustas. O CRISPR/Cas9 é promissor, mas permanece em fase pré-clínica.^7^

Anteriormente, se acreditava que a prevalência da HP1 era de cerca de 1:1.000.000, mas estudos recentes mostram que sua prevalência pode estar por volta de 1:58.000.^10^ Também, se estima que as HPs estão presentes em cerca de 7-14% das crianças com nefrocalcinose,^10^ destacando a importância de um maior alerta acerca desta patologia.

## Conclusão

Devido à raridade e aos fenótipos heterogêneos, a HP1 é frequentemente não reconhecida, resultando em atrasos no diagnóstico e alta morbimortalidade. Esta série de casos descreve irmãos com oxalose sistêmica e diferentes níveis de acometimento cardíaco, incluindo achados de imagem que demonstram a oxalose como cardiomiopatia infiltrativa, como mostrado no caso 1, cuja análise post-mortem do miocárdio confirmou o envolvimento cardíaco. A literatura sugere que condições cardíacas infiltrativas com redução de FE aumentam o risco de tromboembolismo, como observado no caso 1. Sugere-se, então, que médicos de pacientes com HP1 mantenham vigilância rigorosa sobre o status cardíaco, especialmente se a função renal estiver afetada. Além disso, a avaliação de oxalose deve ser feita assim que o diagnóstico de HP1 for concretizado.

## References

[1] Cochat P, Rumsby G (2013). Primary Hyperoxaluria. N Engl J Med.

[2] Fargue S, Bourdain CA (2022). Primary Hyperoxaluria Type 1: Pathophysiology and Genetics. Clin Kidney J.

[3] Sweet ME, Mestroni L, Taylor MRG (2018). Genetic Infiltrative Cardiomyopathies. Heart Fail Clin.

[4] Mookadam F, Smith T, Jiamsripong P, Moustafa SE, Monico CG, Lieske JC (2010). Cardiac Abnormalities in Primary Hyperoxaluria. Circ J.

[5] Riley DS, Barber MS, Kienle GS, Aronson JK, von Schoen-Angerer T, Tugwell P (2017). CARE Guidelines for Case Reports: Explanation and Elaboration Document. J Clin Epidemiol.

[6] Strauss SB, Waltuch T, Bivin W, Kaskel F, Levin TL (2017). Primary Hyperoxaluria: Spectrum of Clinical and Imaging Findings. Pediatr Radiol.

[7] Dejban P, Lieske JC (2022). New Therapeutics for Primary Hyperoxaluria Type 1. Curr Opin Nephrol Hypertens.

[8] Padda IS, Mahtani AU, Patel P, Parmar M (2024). Small Interfering RNA (siRNA) Therapy.

[9] Groothoff JW, Metry E, Deesker L, Garrelfs S, Acquaviva C, Almardini R (2023). Clinical Practice Recommendations for Primary Hyperoxaluria: An Expert Consensus Statement from ERKNet and OxalEurope. Nat Rev Nephrol.

[10] Mandrile G, Pelle A, Sciannameo V, Benetti E, D'Alessandro MM, Emma F (2022). Primary Hyperoxaluria in Italy: The Past 30 Years and the Near Future of a (Not so) Rare Disease. J Nephrol.

