# Supplementary material for: Hiperoxalúria Primária com Acometimento Cardíaco: Uma Série de Casos
Source: Arq Bras Cardiol. 2026 Apr 1;123(3):e20250574. [Article in Portuguese] doi: 10.36660/abc.20250574 (PMC13128217; doi:10.36660/abc.20250574)
Supplement: Translation

# Translation: Primary Hyperoxaluria With Cardiac Involvement: A Case Series

**DOI:** 10.36660/abc.20250574i

**Authors:** Diane Xavier de Ávila, Christine Zomer Dal Molin, Josué dos Santos Barbosa, Laís Corrêa de Carvalho, João Padula Rocha, Maria Júlia Hallack Moura, Davi Orli Machado Grüdtner, Raquel Bittencourt

**Affiliations:** 1 Brazil Complexo Hospitalar de Niterói, Niterói, RJ – Brazil; 2 Brazil Universidade Federal de Santa Catarina, Araranguá, SC – Brazil; 3 Brazil Serviço de Verificação de Óbitos de Criciúma, Criciúma, SC – Brazil

**Keywords:** Cardiovascular Diseases, Chronic Kidney Failure, Primary Hyperoxaluria

## Introduction

Primary Hyperoxaluria type 1 (PH1) is a rare autosomal recessive disorder caused by AGXT gene mutations, which encodes the enzyme responsible for metabolizing glyoxylate. This dysfunction leads to glyoxylate accumulation and its conversion to oxalate. This results in elevated oxalate levels, tissue deposition, and organ damage, particularly the kidneys and heart.^1^Cardiac involvement may progress to heart failure (HF) and fatal arrhythmias.^3 , 4^An overview of the pathophysiology of PH1 is illustrated in Figure 1 . This report presents two PH1 patients with oxalosis and its cardiac complications. This case series was prepared following the CARE guidelines,^5^submitted and approved by the Ethics Committee of Federal University of Santa Catarina.

**Figure 1:** – PH1 pathophysiology and manifestations.

## Case 1

A 19-year-old Caucasian female, previously diagnosed with bilateral medullary nephrocalcinosis at the age of three, presented with uremic syndrome, anemia, depressed level of consciousness, and intense asthenia. Renal replacement therapy (RRT) was initiated. Hyperoxaluria and prior nephrocalcinosis raised suspicion for PH1. Genetic analysis confirmed biallelic AGXT variants: c.33del and p.Lys12Argfs*34.

Patient’s treatment was limited to a conservative approach, which includes HF management, hemodialysis, and pyridoxine. Lumasiran was unavailable via the public health system. Electrocardiographic findings revealed sinus rhythm with left atrial (LA) overload and left anterior superior divisional block. Echocardiogram showed dilated heart chambers, with prominent biatrial enlargement and eccentric left ventricular (LV) hypertrophy resulting in a systolic dysfunction characterized by a reduced LV ejection fraction (EF) (34,9%, Simpson), with a posterior wall thickness of 13 mm. Imaging studies also found aortic, mitral, and tricuspid valves with mild regurgitation. The concentration of B-type natriuretic peptide (BNP) was over 5000 pg/ml. Agatston score was zero, cardiac magnetic resonance showed diffuse myocardial hypokinesia, severe left systolic dysfunction with LVEF of 28%, moderate right systolic dysfunction with right ventricular (RV) EF of 25%, elevated T1 map values, and absence of atrial thrombus. These findings, especially the echocardiographic findings, were suggestive of infiltrative cardiomyopathy, probably related to this systemic disease.

One year after beginning RRT, the patient experienced sudden death while awaiting a medullary biopsy in the hospital, and despite the prompt initiation and effective cardiopulmonary resuscitation (CPR), the patient was declared deceased. An autopsy was performed, indicating pulmonary thromboembolism as the primary cause of death, possibly secondary to underlying cardiac dysfunction. Histopathology showed myocardial atrophy and oxalate deposits in myocytes, intravascular, and interstitial areas. Such deposits were also evidenced in the pericardium and endocardium, with no inflammation ( Figures 1 and 2 ).

**Figure 2:** – Myocardial tissue with oxalosis. H&E staining (40x).

## Case 2

A 42-year-old male, brother of the previously described patient, had no prior history of kidney disease until the age of 35, when he required the initiation of RRT due to extreme levels of nitrogenous wastes, anemia, and hyperparathyroidism. Imaging exams revealed a right kidney with reduced dimensions, left kidney enlargement, and hydronephrosis secondary to a ureterolithiasis, bilateral kidney stones, and loss of corticomedullary differentiation. The DMSA renal scan revealed severe chronic impairment of tubular renal function. Due to the family history, the PH1 diagnosis was suspected, and a genetic test revealed both pathogenic mutations in the AGXT gene: c.33del (p.Lys12Argfs*34) and c.508G>A (p.Gly170Arg). Further cardiac investigations showed diastolic dysfunction of the LV, preserved LV systolic function (74,9%, Simpson), as well as right atrial (RA) enlargement and no evidence of valvular dysfunction. Concentration of BNP was 875 pg/ml, and serum oxalate previous to treatment was 59 µml/ml. About 2 years later, the patient obtained legal authorization to start treatment with lumasiran, covered by the SUS (Brazilian public health system).

## Discussion

PH1 is the most common and severe form of primary hyperoxalurias (PHs). PH1 is an autosomal recessive disorder particularly prevalent in North Africa and the Middle East, given the high rate of consanguineous marriages, and results from a deficiency in the enzyme AGT encoded by AGXT. Over 200 AGXT mutations exist, notably p.Gly170Arg and c.33dupC.^1^

Even though PH1 diagnosis is strongly considered in patients with recurrent calcium oxalate stones, nephrocalcinosis, progressive chronic kidney disease (CKD) of unknown etiology, or a family history of stones and CKD, factors such as the heterogeneous presentation, the nonspecific symptoms, and the reliance on genetic testing for confirmation lead to a challenging diagnosis. Up to 50% of PH1 cases are diagnosed at advanced CKD stages, increasing mortality.^1^However, screening relatives can aid early diagnosis and makes possible a successful treatment.^1^

The variability in age of onset and disease severity is an additional challenge in identifying PH1. The onset of the disease may occur at all ages, but it typically manifests in childhood with nephrolithiasis, nephrocalcinosis, and renal failure.^1^As soon as the diagnosis is made, assessment of systemic oxalosis should be mandatory.

Urinary calcium oxalate supersaturation leads to nephrolithiasis and nephrocalcinosis. As renal function declines, oxalate clearance diminishes, causing systemic deposition, notably cardiac.^3 , 4^Other affected organs include bones, vessels, liver, spleen, skin, and glands.^6^

This physiopathology led to the cardiac abnormalities of case 1, which were in accordance with a previous case series description.^4^In the cited retrospective review, the following alterations were observed: LV hypertrophy, bundle branch block, atrioventricular block, valve dysfunction, impaired EF, large LV mass index, LA enlargement, impaired RV function, pulmonary hypertension, diastolic dysfunction, and infiltrative processes. All of these alterations predispose to HF and fatal arrhythmias. In the referenced study, people with cardiac abnormalities had a creatinine mean of 7.16 (0.9-33.2) mg/dL, while people with normal hearts had a mean of 1.93 (0.3-21.6) mg/dL. Moreover, 77.8% of people with heart alterations had end-stage kidney disease.^4^

These data highlight the need to monitor cardiac function in PH1, particularly in renal impairment. Early oxalate reduction may prevent cardiac involvement, stressing the need for access to modern treatments, which will be discussed in the following section.

Two medications for the management of PH1 have recently gained prominence: Lumasiran and Nedosiran.^7^Case 1 patient’s treatment was limited to a conservative approach, but the case 2 patient has been under lumasiran treatment for the past 9 months.

Despite the high cost of these medications, they significantly reduce the production of oxalate. Lumasiran and Nedosiran inhibit hepatic oxalate production through RNA interference therapy. The main adverse events of these pharmacals are injection site reactions (Lumasiran and Nedosiran) and abdominal pain/discomfort (Lumasiran),^8^but case 2 experienced no side effects.

Another approach to PH1 is pyridoxine, for which responsiveness is recommended to be tested in all PH1 patients (strong recommendation).^9^Other therapies, including stiripentol, lanthanum carbonate, and Oxalobacter formigenes, lack robust evidence. CRISPR/Cas9 is promising but remains preclinical.^7^

PH1 is more common than once thought. Previously, it was believed that the prevalence was around 1:1.000.000, but recent studies point out that its prevalence may be around 1:58.000.^10^Moreover, PHs are estimated to be present in 7-14% of children with nephrocalcinosis,^10^highlighting the need for greater clinical awareness.

**Figure 3:** – Myocardial tissue with oxalosis. H&E staining (40x).

## Conclusion

Based on its rarity and heterogeneous phenotype, PH1 is often underrecognized, resulting in delayed diagnosis and high morbidity and mortality. This case series described siblings with systemic oxalosis and varying degrees of cardiac involvement, highlighting imaging features consistent with infiltrative cardiomyopathy with restrictive physiology, and, as shown in case 1, postmortem myocardial analysis confirmed cardiac involvement. The literature suggests that infiltrative cardiac conditions with impaired ventricular function have an increased risk of thromboembolism, as observed in case 1. We suggest that physicians caring for patients with PH1 should pay close attention to cardiac status, especially if renal function is impaired, and oxalosis screening should be promptly evaluated as soon as the diagnosis of PH1 is confirmed.
